# Synthesis and Evaluation of Bithiazole Derivatives As Potential α-Sarcoglycan Correctors

**DOI:** 10.1021/acsmedchemlett.3c00046

**Published:** 2023-07-28

**Authors:** Giovanni Ribaudo, Marcello Carotti, Alberto Ongaro, Erika Oselladore, Martina Scano, Giuseppe Zagotto, Dorianna Sandonà, Alessandra Gianoncelli

**Affiliations:** † Department of Molecular and Translational Medicine, University of Brescia, viale Europa 11, 25121 Brescia, Italy; ‡ Department of Biomedical Sciences, University of Padova, via Ugo Bassi 58/B, 35131 Padova, Italy; § Department of Pharmaceutical and Pharmacological Sciences, University of Padova, via Marzolo 5, 35131 Padova, Italy

**Keywords:** bithiazole, α-sarcoglycan, CFTR, myotubes

## Abstract

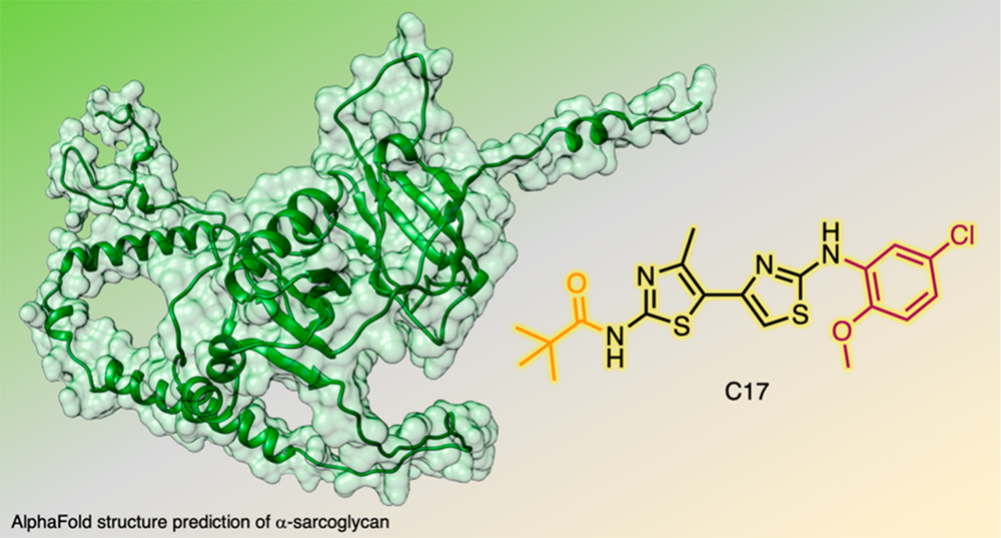

4′-Methyl-4,5′-bithiazoles were previously identified as cystic fibrosis transmembrane regulator (CFTR) correctors, thus being able to correct folding defective mutants of the channel regulating chloride transport through the membrane. Additionally, bithiazole derivative **C17** was reported to recover α-sarcoglycan *in vitro* and *in vivo*. We report here the synthesis of two new derivatives of **C17**, in which the two sides of the bithiazole scaffold were modified. The synthesized compounds and the corresponding precursors were tested in myogenic cells to evaluate the expression of α-sarcoglycan. The results highlighted that both substitutions of the bithiazole scaffold are important to achieve the maximum recovery of the α-sarcoglycan mutant. Nonetheless, partial preservation of the activity was observed. Accordingly, this paves the way to further derivatizations/optimization and target fishing studies, which were preliminarily performed in this study as a proof of concept, allowing the investigation of the molecular mechanisms leading to the α-sarcoglycan correction.

Thiazole is a five-membered ring bearing two heteroatoms, consisting of one sulfur atom and one nitrogen atom at positions 1 and 3. Compounds based on this scaffold show diverse biological activities translated into molecules of pharmacological interest.^1^

Antibacterial, antifungal, antiparasitic, antiulcer, anti-inflammatory, and antiproliferative effects are only a portion of reported effects for thiazole-based small molecules. Additionally, compounds bearing two thiazole rings, separated by linkers or by a single bond such as bleomycin, have been approved as antiviral, antibacterial, and anticancer agents.^2^ This class of compounds is constantly attracting the interest of medicinal chemists, and derivatives bearing two thiazole rings were recently reported to show multitarget anticancer activity.^3^

In previous reports, 4′-methyl-4,5′-bithiazoles were identified as cystic fibrosis transmembrane regulator (CFTR) correctors, i.e., compounds able to correct some folding defective mutants of the channel responsible for the control of chloride transport across the plasma membrane of many cell types.^4^ Structure–activity relationship (SAR) studies were conducted through the introduction of different substituents in this scaffold, guiding the identification of most the substituents leading to optimal bioactivity.^5^ In this respect, a compound named **C17** emerged as one of the most promising lead compounds (Figure 1A).^5−8^

**Figure 1:**
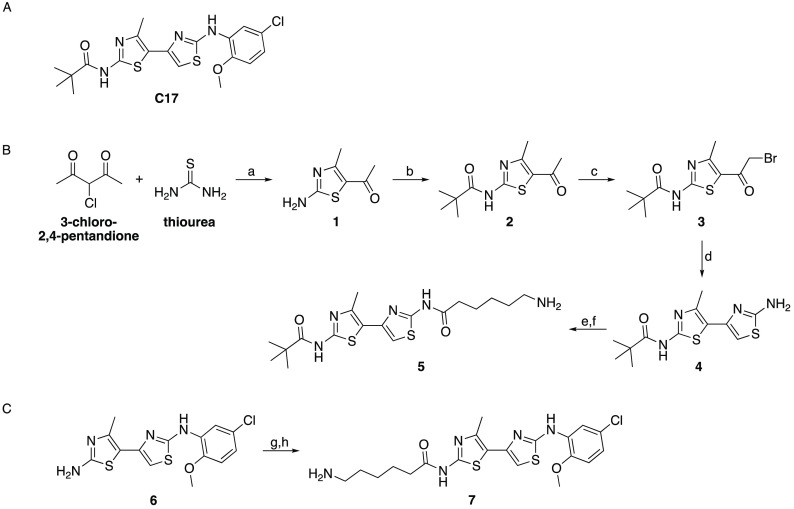
(A) Chemical structure of **C17**. (B) Synthetic scheme and reaction conditions for the preparation of compound **5**: (a) ethanol; (b) pivalic acid, DCC, DMAP, dichloromethane; (c) HBr, acetic acid, pyridinium tribromide; (d) thiourea, ethanol; (e) 6-(Boc-amino)hexanoic acid, CDI, DMF; (f) TFA, dichloromethane. (C) Synthetic scheme and reaction conditions for the preparation of compound **7**: (g) 6-(Boc-amino)hexanoic acid, DMF, DIPEA, TBTU; (h) TFA, dichloromethane.

This derivative was also tested for its ability to recover folding defective mutants of proteins other than CFTR such as the I661T mutant of ATP8B1, a protein belonging to the ABC transporter family that lacks homology with CFTR.^9^ More recently, **C17** efficacy was evidenced in *in vitro* and *in vivo* models of the limb girdle muscular dystrophy R3 (LGMDR3), a rare genetic disease caused by mutations of the α-sarcoglycan protein.^10,11^ In both heterologous cell models and LGMDR3 myogenic cells, derived from a subject carrying missense mutations on the α-sarcoglycan coding gene, the salvage of the mutated protein in terms of quantity and localization was observed. Notably, in the humanized mouse model expressing the R98H-α-sarcoglycan, recovery of the defective protein corresponded to improvement in muscle strength.^12−14^ Thus, the 4′-methyl-4,5′-bithiazole derivatives, first screened for the treatment of cystic fibrosis, seemed to hold a promiscuous action, being able to recover proteins, different in terms of structure, but sharing with CFTR a similar fate when carrying missense mutations. Because of this feature, **C17** and derivatives cover an interesting role in the field of protein misfolded diseases that surely deserves to be further studied, also identifying their specific interactor(s) and providing optimized compounds.

In this context, more recent studies aimed at investigating the efficacy of constrained bithiazoles that showed higher maximum efficacy in cystic fibrosis cells, however without significant enhancement of potency.^8,15^ Additionally, Martina et al. very recently reported a SAR study based on bithiazole derivatives inspired by CFTR correctors aiming at the identification of multifunctional antibacterial agents.^16^

Here, the scaffold of the corrector **C17** has been modified, introducing a suitable linker for future resin functionalization useful for chromatographic experiments aiming at the capture of the compound interactor(s),^17^ and the activity of the obtained compounds in influencing α-sarcoglycan expression by myotubes was tested.

In this work, we describe the synthesis of two previously undisclosed derivatives of **C17**, in which the substituents on the two sides of the bithiazole scaffold were alternatively substituted with a linker terminating with an amino group.

The preparation of compound **5** was based on the procedure reported by Davison et al. for steps a–d (Figure 1B), with small modifications.^18^ Briefly, 3-chloro-2,4-pentandione was reacted with thiourea to provide compound **1**, which was converted to compound **2** upon reaction with pivalic acid. Bromination with HBr, acetic acid, and pyridinium tribromide afforded compound **3** and another reaction with thiourea led to the isolation of compound **4**. Compound **5** was then obtained by reaction with 6-(Boc-amino)hexanoic acid, and the intermediate was then deprotected through treatment with TFA to give the final compound. Preparation of compound **7** was straightforward (Figure 1C) and was achieved by reaction of the commercially available intermediate **6** with 6-(Boc-amino)hexanoic acid, followed by deprotection with TFA. The final compounds were characterized by NMR, mass spectrometry, and HPLC (Figures S1–S8 in the Supporting Information). The synthesis of compound **7** can also be achieved through the Fmoc protection strategy, starting from derivatization of compound **6** with 6-(Fmoc-amino)hexanoic acid, but lower yields were obtained in our attempts (data not shown).

The amino moieties introduced with this synthesis could be exploited for further derivatizations or for the immobilization of the ligands in the context of ligand–target interaction studies, such as those based on surface plasmon resonance (SPR), affinity chromatography, or probing/labeling, thus assessing the contribution of the different portions of **C17** to the biological activity to aid the investigation of the underlying molecular mechanisms.

For **5** and **7**, relevant physicochemical descriptors were calculated using SwissADME, and the drug-likeness of the synthesized compounds was preliminarily assessed.^19,20^ Overall, compounds **5** and **7** can be defined as druglike molecules, even if slightly high flexibility and polarity values were computed when compared to ideal features for pharmacokinetic parameters (Figure S9 in the Supporting Information). Moreover, based on the computed physicochemical descriptors, the compounds are predicted to be partially absorbed through the gastrointestinal tract, without crossing the blood–brain barrier.

Thus, we tested compounds **4**, **5**, **6**, and **7** in the myogenic cells described above, comparing their effectiveness with the one of the lead compounds, **C17**. After treatment for 72 h, cells were lysed, proteins were resolved by SDS PAGE, and the content of α-sarcoglycan was analyzed by Western blot (WB; Figure 2).

**Figure 2:**
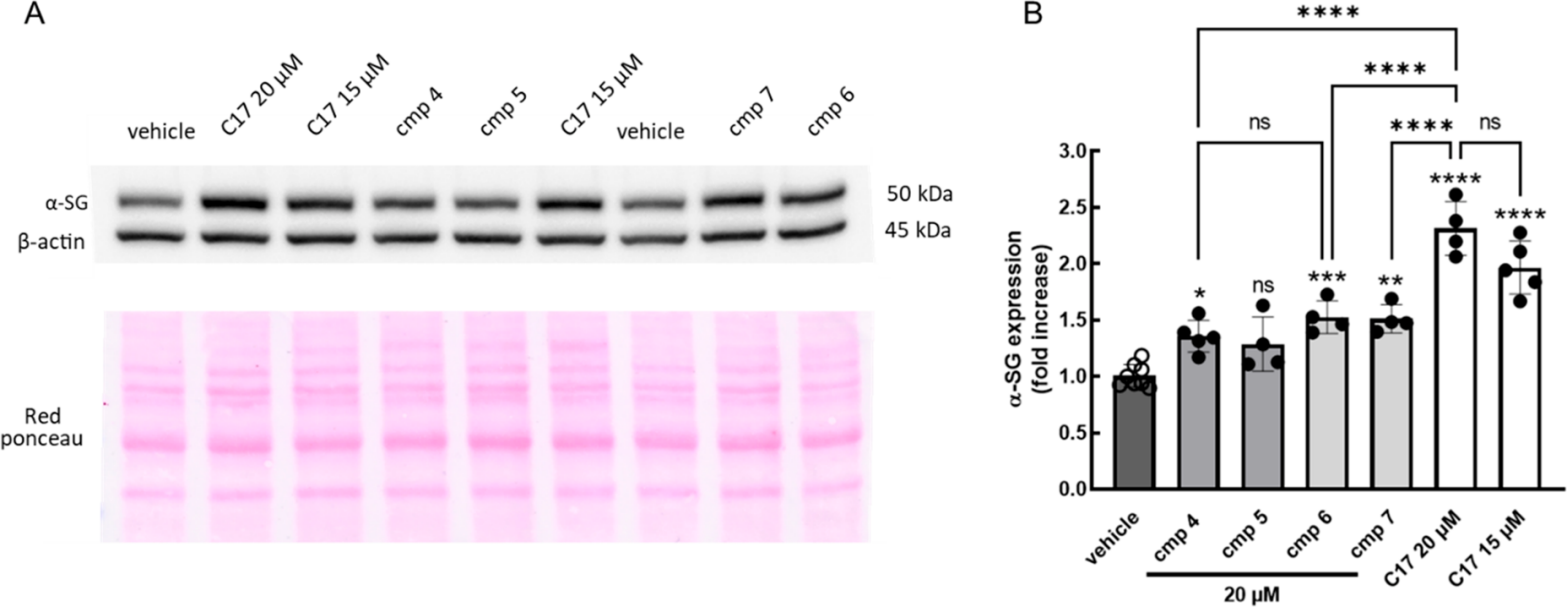
(A) Representative Western blot for the analysis of the α-sarcoglycan (α-SG) content in cells from a LGMDR3 subject carrying the L31P/V247 M α-SG mutations. Cells were differentiated for 7 days and treated for the last 72 h with either 1‰ DMSO (vehicle), the different compounds (cmp), or **C17** at the indicated concentrations. Both anti α-sarcoglycan and β-actin antibodies were used, the last one for normalizing the content of loaded proteins. The red ponceau staining of the membrane is reported for reference. (B) The graph reports the densitometric analysis of independent WB experiments. The α-SG content is expressed as the fold increase of the amount present in the vehicle treated myotubes, the mean value ± SD of a minimum four independent experiments, each one done in duplicate/triplicate, is also reported. Statistical analysis was performed by a one-way ANOVA test followed by multiple comparison Tukey’s test; ns, *p* > 0.05, *p ≤ 0.05, **p ≤ 0.01, ***p ≤ 0.00, ****p ≤ 0.0001. Boc- and Fmoc-protected precursors of compound **7** were also tested, and the results are reported in Figure S10 in the Supporting Information.

Preliminary SAR information can be deduced from the results of the experiments. As expected, **C17** at both 20 and 15 μM (the concentration used in all previous experiments), elicited the maximum effect, doubling the content of the mutated α-sarcoglycan expressed by myotubes.^12,13^ The novel chemical entities maintained part of the activity, even though lacking either the substituted phenyl moiety, in the case of compound **4**, or the *tert*-butyl moiety, in the case of compound **6**. Indeed, the α-sarcoglycan rescue was significantly higher than the control, consisting of cells treated with the vehicle, both globally (Figure 2) and when the membrane fraction was analyzed (Figure S11 in the Supporting Information) for both molecules. It must be noted that the tested compounds were demonstrated to be less effective than the original molecule **C17**, suggesting that both modifications are detrimental. Nevertheless, data on a wider set of compounds bearing modifications in terms of chain length, composition, position on the scaffold, and ramification or substitution would be needed to fully assess the significance in terms of the SAR of such variations.

On the other hand, the presence of the long hydrophobic chain in compound **7** did not affect activity drastically, and this holds true also for other precursors of compound **7** bearing the same hydrophobic chain that were studied, as reported in the Supporting Information. This is an ideal condition for the potential application of the compounds for the functionalization of resins in the context of target fishing studies, where the long hydrophobic chain can serve to pull the active part of the molecule away from the beads, thereby reducing steric hindrance during the binding of interactors.

In this context, a preliminary target fishing experiment, carried out using beads functionalized with compound **5** and compound **7** following a protocol reported in the literature,^17,21^ evidenced the ability of both derivatized resins to recover several protein interactors (Figure S12 in the Supporting Information). This experiment was intended as a proof of concept, and notably, some of the proteins were clearly displaced when the resins were incubated with an excess of free **C17** molecule before the elution step, promisingly suggesting the presence of specific **C17** interactors among the retrieved proteins.

In conclusion, according to these results, it is possible to infer that, besides the bithiazole core, both substituted moieties should be present to guarantee the maximum recovery of the α-sarcoglycan mutant. On the other hand, the preservation of part of the activity of the original molecule allows us to be confident that these derivatives may serve as useful tools for the identification of possible **C17** interactors in target fishing experiments and for the clarification of its mechanism of action in sarcoglycanopathy.

Overall, this information is of major importance for the possibility of derivatizing **C17**, paving the way for mechanistic studies, and guiding further optimization of the compound.

## Experimental Procedures

### Chemistry

Commercially available chemicals were purchased from Sigma-Aldrich and used without further purification. NMR experiments were performed on Bruker Avance III and Bruker Ascend spectrometers (frequencies: 400.13 and 100.62 MHz for ^1^H and ^13^C, respectively; Bruker, Billerica, MA). For data processing, TopSpin 4.1.4 and iNMR 6.4.5 (Nucleomatica, Molfetta, Italy) were used, and the spectra were calibrated using a solvent signal. Mass spectra were recorded by direct infusion electrospray (ESI) on an LCQ Fleet ion trap mass spectrometer (Thermo Fisher Scientific, Waltham, MA) and on a Xevo G2 QTof high-resolution mass spectrometer (HRMS; Waters, Milford, MA). For data processing, the Qual Browser Thermo Xcalibur 4.0.27.13 software was used. The purity profile was assayed by HPLC using a Pro-Star system (Palo Alto, CA) equipped with a 1706 UV–vis detector (Bio-Rad, Hercules, CA) and a C-18 column (5 μm, 4.6 × 150 mm; Agilent, Santa Clara, CA). An appropriate gradient of 0.1% formic acid (A) and acetonitrile (B) was used as the mobile phase with an overall flow rate of 1 mL min^–1^. The general methods for the analyses are reported in the following.

Method for compound **5**: 0 min (90% A–10% B), 1 min (90% A–10% B), 9 min (5% A–95% B), 12 min (5% A–95% B), and 13 min (90% A–10% B). Method for compound **7**: 0 min (90% A–10% B), 2 min (90% A–10% B), 10 min (5% A–95% B), 14 min (5% A–95% B), and 16 min (90% A–10% B). Analyses were performed at 254 nm, and the purity profile was above 95% for final compounds (area %). Detailed synthetic procedures are described in the Supporting Information. Throughout the experiments, no unexpected or unusually high safety hazards were encountered. In the Supporting Information, ^1^H NMR, ^13^C NMR, ESI-MS spectra, and HPLC chromatograms are reported for final compounds (Figures S1–S8).

### Prediction of Physicochemical Descriptors

Physicochemical descriptors considered to be relevant for drug-likeness, ADME parameters, and pharmacokinetic properties were predicted for compounds **5** and **6** using SwissADME (www.swissadme.ch, accessed on November 1, 2022, Molecular Modeling Group - Swiss Institute of Bioinformatics, Lausanne, CH).^19,20^ Results were plotted as radar graphs reporting the ideal chemical space for oral bioavailability, according to lipophilicity, size, polarity, solubility, insaturation, and flexibility scores of the molecules.

### Cell Growth, Differentiation, and Treatments

Primary myogenic cells from a LGMDR3 subject (compound heterozygote for the V247 M and L31P mutations in the α-sarcoglycan protein)^22^ were grown in proliferation medium (PM) composed by DMEM (Sigma-Aldrich, St. Louis, MO, USA), at pH 7.2 supplemented with 30% Foetal Bovine Serum (FBS, Thermo Fisher Scientific,Waltham, MA, USA), 10 μg/mL insulin, Fibroblast Growth Factor (FGF, 25 ng/mL), and Epidermal Growth Factor (EGF, 10 ng/mL; Sigma-Aldrich, St. Louis, MO, USA). Myotube differentiation was induced by replacing PM with differentiating medium (DM) composed of DMEM supplemented with 2% Horse Serum (Euroclone, Milano,Italy), 10 μg/mL human recombinant insulin (Sigma, St. Louis, MO, USA), and 100 μg/mL human transferrin (Sigma-Aldrich, St. Louis, MO, USA). Differentiation was carried out for 7 days. Treatments with compounds were carried out during the last 72 h of differentiation by the addition of vehicle (DMSO 1 ‰) or the different compounds dissolved in DMSO (final concentration 1‰). At the end of the treatments, myotubes were washed twice with ice-cold PBS and then lysed with RIPA buffer (Tris-HCl 50 mM at pH 7.5, NaCl 150 mM, NP-40 1% v/v, sodium deoxycholate 0.5% w/v, SDS 0.1% w/v) supplemented with complete protease inhibitor cocktail (Sigma-Aldrich, St. Louis, MO, USA).

### Western Blot Analysis

Protein concentration in total protein lysates was determined with a BCA assay (Thermo Fisher Scientific, Waltham, MA, USA) according to the manufacturer’s instructions. Equal amounts of total proteins were separated by SDS-PAGE and transferred to a nitrocellulose membrane by semidry blotting. Membranes were blocked with 5% w/v skim milk in TBS-T buffer and probed with rabbit monoclonal anti-α-sarcoglycan (AB189254 from Abcam, Cambridge, UK) and mouse monoclonal anti-β-actin (Sigma-Aldrich, St. Louis, MO, USA); secondary antibodies were horseradish peroxidase-conjugated. Blots were developed with ECL chemiluminescent substrate (Euroclone, Milano, Italy), and chemiluminescent signals were digitally acquired with the Alliance Mini HD9 Imaging System (Uvitec, Cambridge, UK). Densitometry was performed with ImageJ software. The intensities of the α-sarcoglycan bands were normalized for the intensity of the Ponceau Red staining of the total protein loaded and for the intensity of the β-actin signal.

### Biotinylation of Surface Proteins

For the biotinylation reaction, at the end of the treatment, myotubes were incubated at 4 °C for at least 10 min, and all of the procedures were performed at this temperature to slow down cellular processes preventing the internalization of biotin. Cells were then washed three times with ice cold PBS supplemented with calcium and magnesium and incubated under gentle agitation with a solution of 0.5 mg/mL biotin (EZ-Link Sulfo-NHS-LC-Biotin, Thermo Fisher) in PBS for 20 min at 4 °C. The biotinylation reaction was stopped, washing cells twice with 100 mM glycine in PBS (each wash 5 min) and twice with PBS. Cells were lysed with RIPA buffer, and lysates were centrifuged 30 min at 20 000*g* at 4 °C. The supernatants were quantified by BCA assay, and 50 μg of proteins were incubated with streptavidin agarose resin (Thermo Fisher, 30 μL of resin for each sample) overnight at 4 °C. The streptavidin resin, after the incubation under gentle rotation with myotube lysates, was washed three times with RIPA buffer. Bound proteins were eluted with Laemmli sample buffer and analyzed by Western blot. The absence of biotin internalization was assessed by probing the Western blot membranes with an antibody specific for the cytosolic protein β-actin.

### Statistical Analysis

Values are expressed as means ± SD; statistical differences among groups were then determined by a one-way ANOVA test, followed by either Tukey’s test for simultaneous multiple comparisons or a Dunnet test for simultaneous multiple comparisons with a control. A level of confidence of *p* < 0.05 was considered for statistical significance.

## Supporting Information Available

Synthetic procedures, NMR and ESI-MS spectra, HPLC chromatograms, results of computational studies, and additional Western blot data (PDF)
