# Supplementary material for: Synthesis and Evaluation of Bithiazole Derivatives As Potential α-Sarcoglycan Correctors
Source: ACS Med Chem Lett. 2023 Jul 28;14(8):1049–53. doi: 10.1021/acsmedchemlett.3c00046 (PMC10424318; doi:10.1021/acsmedchemlett.3c00046)
Supplement: Supplementary file 1 — ml3c00046_si_001.pdf [file ml3c00046_si_001.pdf]

# - Supporting Information -

Synthesis and evaluation of bithiazole derivatives as potential α-sarcoglycan correctors

Giovanni Ribaudo[1], Marcello Carotti[2], Alberto Ongaro[3], Erika Oselladore[1], Martina Scano[2], Giuseppe Zagotto[3], Dorianna Sandonà[2*] and Alessandra Gianoncelli[1*]

[1] Department of Molecular and Translational Medicine, University of Brescia, viale Europa 11, 25121 Brescia, Italy

[2] Department of Biomedical Sciences, University of Padova, via Ugo Bassi 58/B, 35131 Padova, Italy

[3] Department of Pharmaceutical and Pharmacological Sciences, University of Padova, via Marzolo 5, 35131 Padova, Italy

*Corresponding authors: Prof. Dorianna Sandonà, Department of Biomedical Sciences, University of Padova, via Ugo Bassi 58/B, 35131 Padova, Italy, dorianna.sandona@unipd.it; Prof. Alessandra Gianoncelli, Department of Molecular and Translational Medicine, University of Brescia, viale Europa 11, 25121 Brescia, Italy, alessandra.gianoncelli@unibs.it

Content

Synthetic procedures 3

NMR, ESI-MS and HPLC characterization of final compounds 5

Figure S1. ^{1}H-NMR of compound 5 (MeOD). 5

Figure S2. ^{13}C-NMR of compound 5 (MeOD). 5

Figure S3. ESI-MS spectrum of compound 5 (positive ionization mode). 6

Figure S4. HPLC-UV profile (254 nm) of compound 5. 6

Figure S5. 1H-NMR of compound 7 (DMSO-d_6). 7

Figure S6. ^{13}C-NMR of compound 7 (DMSO-d_6). 7

Figure S7. ESI-MS spectrum of compound 7 (positive ionization mode). 8

Figure S8. HPLC-UV profile (254 nm) of compound 7. 8

Computational studies 9

Figure S9. Overview of the predicted physicochemical descriptors for compounds 5 and 7. 9

Western blot analysis 10

Figure S10. Additional in vitro studies. 10

Figure S11. Analysis of the fraction of α-sarcoglycan (α-SG) present at the membrane of LGMDR3 myogenic cells. 11

Figure S12. PoC of affinity chromatography experiments carried out with functionalized cmp7 and cmp5. 12

Synthetic procedures

Synthesis of 1-(2-amino-4-methylthiazol-5-yl)ethan-1-one (1)

Thiourea (550 mg, 7.22 mmol) and 3-chloro-2,4-pentandione (815 µL, 7.22 mmol) formed a suspension in ethanol (30 mL) which was heated to reflux for 20 hours and monitored by TLC (95% dichloromethane, 5% methanol). The resulting solution was then cooled in ice prompting the formation of crystals. The solid was filtered using a Buchner funnel and washed with cold ethanol followed by ether. Yield: 98%. δ_H (400 MHz, DMSO-d_6): 2.41 (3H, s, -CH_3), 2.48 (3H, s, -$COCH_3$).

Synthesis of N-(5-acetyl-4-methylthiazol-2-yl)pivalamide (2)

Pivalic acid (183 mg, 1.79 mmol), DCC (738 mg, 3.58 mmol) and DMAP (437 mg, 3.58 mmol) were loaded into a round bottomed flask and dissolved in dichloromethane (10 mL). The reaction was stirred at room temperature for 1.5 hours, and compound **1** (300 mg, 1.92 mmol) was then added. The reaction was stirred at room temperature for 36 hours and monitored by TLC (hexane/ethyl acetate, 3:2). The mixture was diluted in dichloromethane (25 mL), washed with basic water (4 x 30 mL) and evaporated under reduced pressure to provide an off-white solid. Yield: 88%. δ_H (400 MHz, acetone-d_6): 1.23 (9H, s, -CH_3), 2.32 (3H, s, -CH_3), 2.42 (3H, s, -$COCH_3$).

Synthesis of N-(5-(2-bromoacetyl)-4-methylthiazol-2-yl)pivalamide (3)

Compound **2** (130 mg, 0.54 mmol) was dissolved in a solution of HBr in acetic acid (33% w/w, 9.1 mL). Then, pyridinium tribromide (194 mg, 0.61 mmol) was added and the reaction was left under stirring at room temperature for 16 hours, and monitored by TLC (hexane/ethyl acetate, 3:1). Reaction mixture was diluted with H_2O (20 mL) and extracted with dichloromethane (3 x 30 mL). Organic layers were combined and washed with basic water (2 x 30 mL). Afterwards, the organic solvent was concentrated under reduced pressure to leave a light brown solid. Yield: 72%. δ_H (400 MHz, acetone-d_6): 1.37 (9H, s, -CH_3), 2.61 (3H, s, -CH_3), 4.45 (2H, s, -$CBrH_2$).

Synthesis of N-(2-amino-4'-methyl-[4,5'-bithiazol]-2'-yl)pivalamide (4)

Compound **3** (120 mg, 0.375 mmol) and thiourea (34 mg, 0.45 mmol) were dissolved in ethanol (8 mL) and heated to reflux. The mixture was left under stirring for 4.5 hours and monitored by TLC (hexane/ethyl acetate, 3:1). The reaction was diluted in H_2O (20 mL) and extracted with ethyl acetate (3 x 30 mL). The crude product, which was obtained by evaporation of the organic solvent, was purified using silica gel column chromatography (hexane/ethyl acetate, 3:2). Yield: 28%. δ_H (400 MHz, DMSO-d_6): 1.25 (9H, s, -CH_3), 2.44 (3H, s, -CH_3), 5.64 (1H, s, -CONH), 6.53 (1H, s, CH), 6.90 (2H, bs, -NH_2).

Sinthesis of 6-amino-N-(2-((5-chloro-2-methoxyphenyl)amino)-4'-methyl-[4,5'-bithiazol]-2'-yl)hexanamide (5)

6-(Boc-amino)hexanoic acid (94 mg, 0.41 mmol) and CDI (67 mg, 0.41 mmol) were dissolved in DMF and stirred at room temperature for 30 minutes. Compound **4** (80 mg, 0.27 mmol) was then added, and the mixture was heated to 100°C and left overnight. The reaction was monitored by TLC (94% dichloromethane, 5% methanol, 1% TEA). Saturated $NaHCO_3$ solution was then added to the mixture and extracted with dichloromethane (3 x 20 mL). The crude Boc-protected product, obtained by the evaporation of the organic solvent, was purified by silica gel chromatography (96% dichloromethane, 3% methanol, 1% TEA). The final compound was obtained by treating the

intermediate with a mixture of 3 mL of dichloromethane and 1 mL of TFA at room temperature for 30 minutes. Solvent was then evaporated to provide the title compound as an off-white solid. Yield: 65%. δ_H (400 MHz, MeOD): 1.3-1.4 (2H, m, -CH_2), 1.422 (9H, s, -CH_3), 1.51 (2H, m, -CH_2), 1.73 (2H, m, -CH_2), 2.48 (2H, m, -CH_2), 2.49 (3H, s, -CH_3), 3.05 (2H, m, -$OCCH_2$), 6.54 (1H, m, NH), 7.01 (1H, s, CH); δ_C (100 MHz, MeOD): 15.7, 24.6, 26.0, 27.4, 29.3, 35.1, 38.8, 39.8, 78.4, 107.6, 121.1, 142.3, 142.8, 156.3, 157.1, 157.2, 157.6, 172.3, 177.2; ESI-MS: calculated for $C_{18}H_{28}N_5O_2S_2^+$ $[M+H]^+$ 410.1679, found 410.1283.

Synthesis of 6-amino-N-(2-((5-chloro-2-methoxyphenyl)amino)-4'-methyl-[4,5'-bithiazol]-2'-yl)hexanamide (7)

6-(Boc-amino)hexanoic acid (63 mg, 0.27 mmol) was dissolved in DMF (500 µL), and DIPEA (60 µL) and TBTU (128 mg, 0.40 mmol) were added. The solution was left to stir for 1 hour at room temperature. Another portion of DIPEA (60 µL) was then added, together with compound **6** (100 mg, 0.28 mmol). The mixture was then left to stir for 16 hours and monitored by TLC (95% dichloromethane, 5% methanol). The reaction was quenched by the addition of 20 mL of water and the compound was extracted using ethyl acetate (3 x 30 mL). Combined organic layers were then washed with deionized H_2O (2 x 50 mL) and evaporated under reduced pressure, providing the Boc-protected final compound as a light green solid. The final compound was obtained by treating the intermediate with a mixture of 3 mL of dichloromethane and 1 mL of TFA at room temperature for 45 minutes. Solvent was then evaporated to provide the title compound as a light green solid. Yield: 68%. δ_H (400 MHz, DMSO-d_6): 1.3-1.4 (2H, m, -CH_2), 1.4-1.6 (4H, m, -CH_2), 2.44 (2H, t, -$NH_2C\underline{H_2}$), 2.52 (3H, s, -CH_3), 2.79 (2H, t, -$OCCH_2$), 3.89 (3H, s, -OCH_3), 6.9-7.1 (3H, m, ArH), 8.68 (1H, s, -ArH), 9.92 (1H, s, NH), 12.06 (1H, s, NH); δ_C (100 MHz, DMSO-d_6): 17.4, 25.6, 25.8, 27.2, 35.1, 39.2, 56.4, 104.6, 112.4, 117.3, 120.5, 121.0, 124.8, 131.7, 142.7, 143.1, 146.8, 155.4, 158.3, 158.7, 163.0, 171.4; ESI-MS: calculated for $C_{20}H_{25}ClN_5O_2S_2^+$ $[M+H]^+$ 466.1133, found 466.1150.

NMR, ESI-MS and HPLC characterization of final compounds

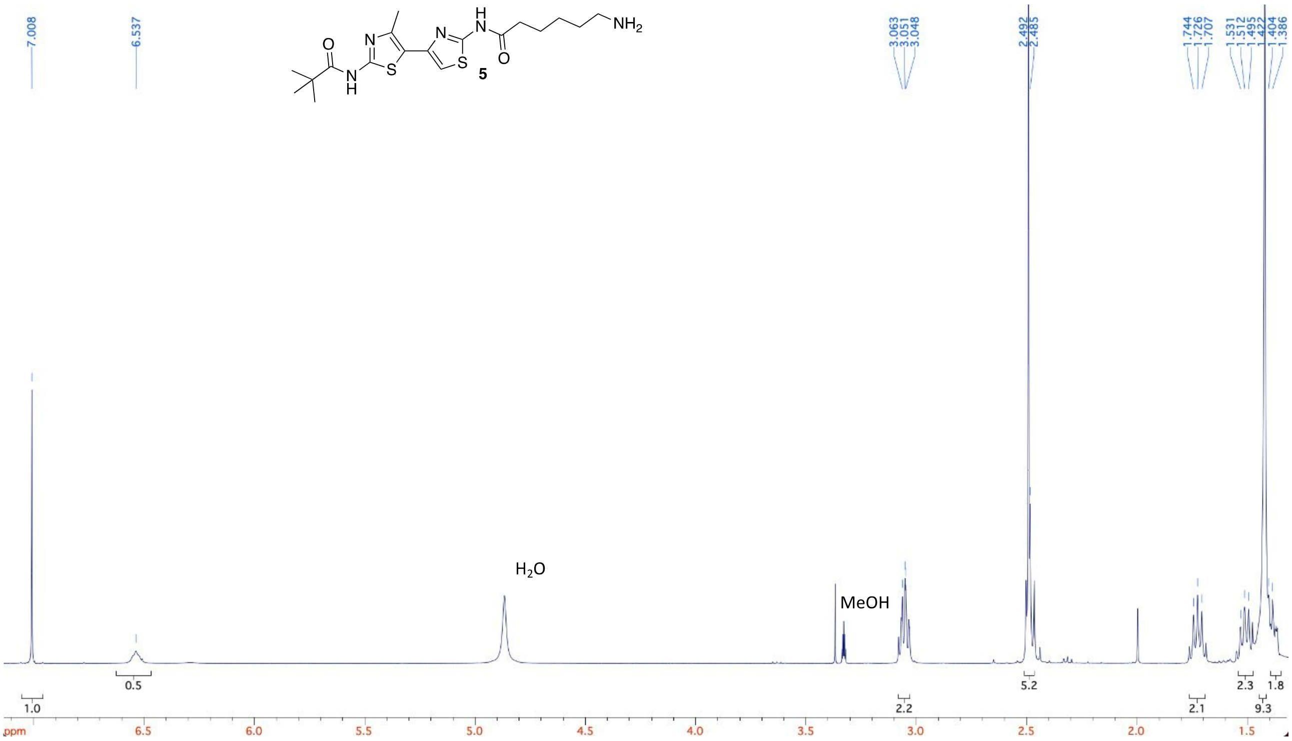

Figure S1. ^{1}H-NMR of compound **5** (MeOD).

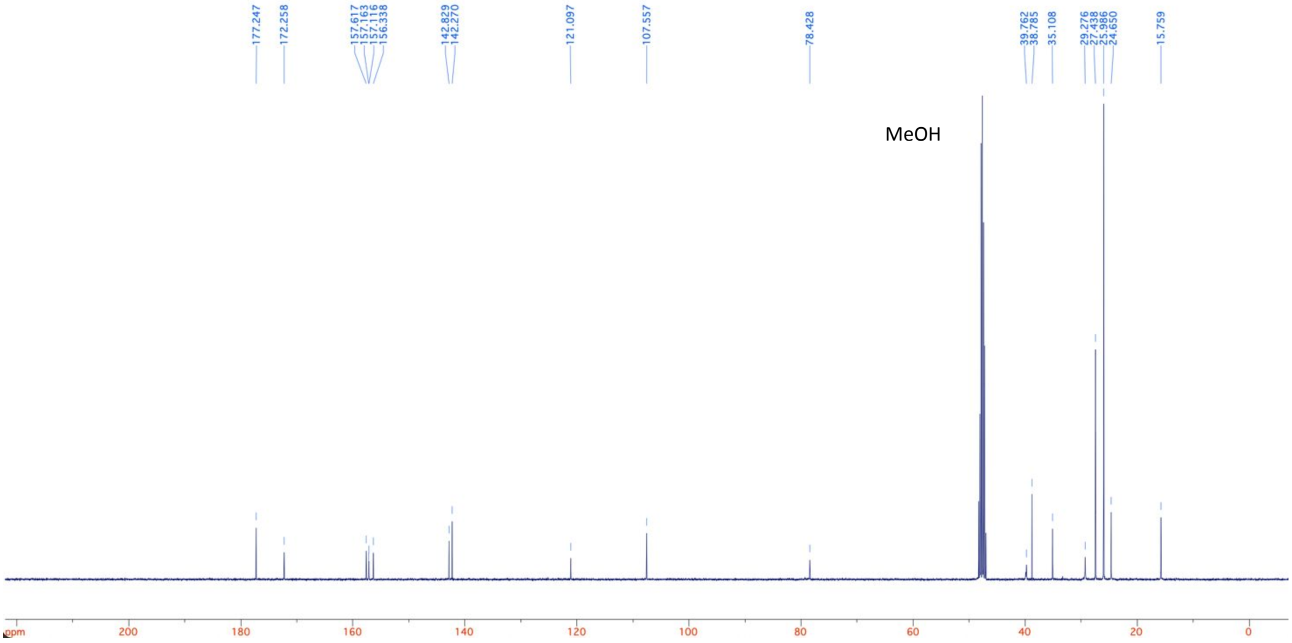

Figure S2. ^{13}C-NMR of compound **5** (MeOD).

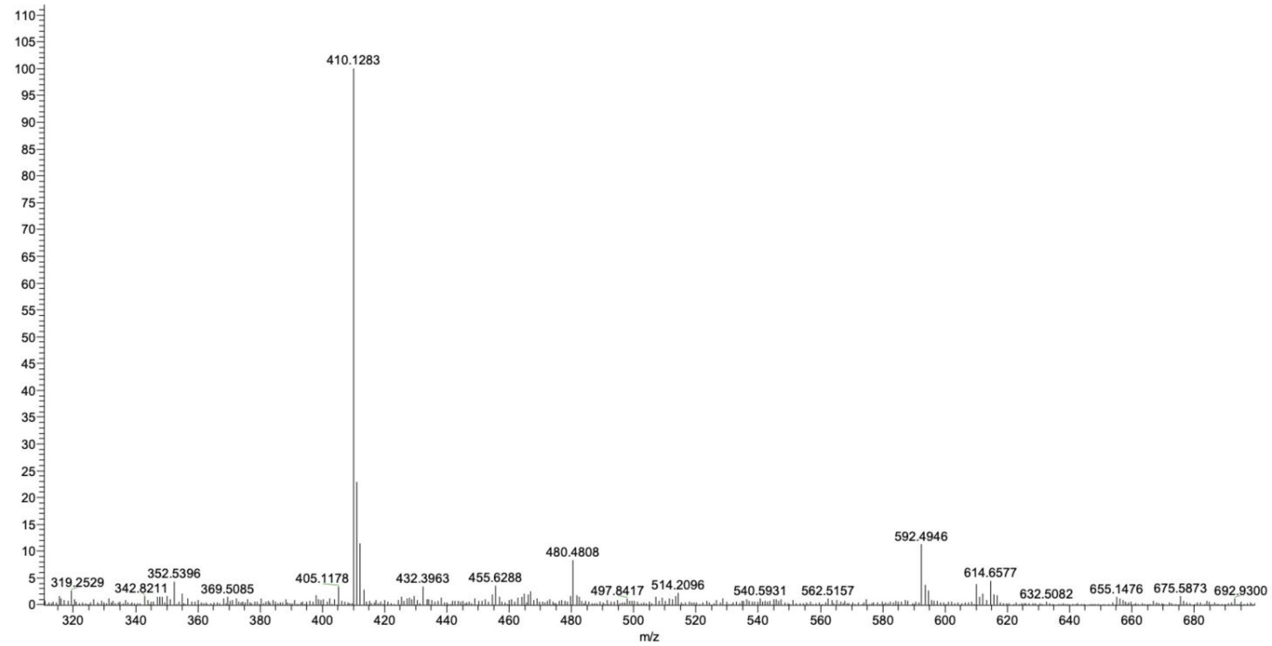

Figure S3. ESI-MS spectrum of compound **5** (positive ionization mode).

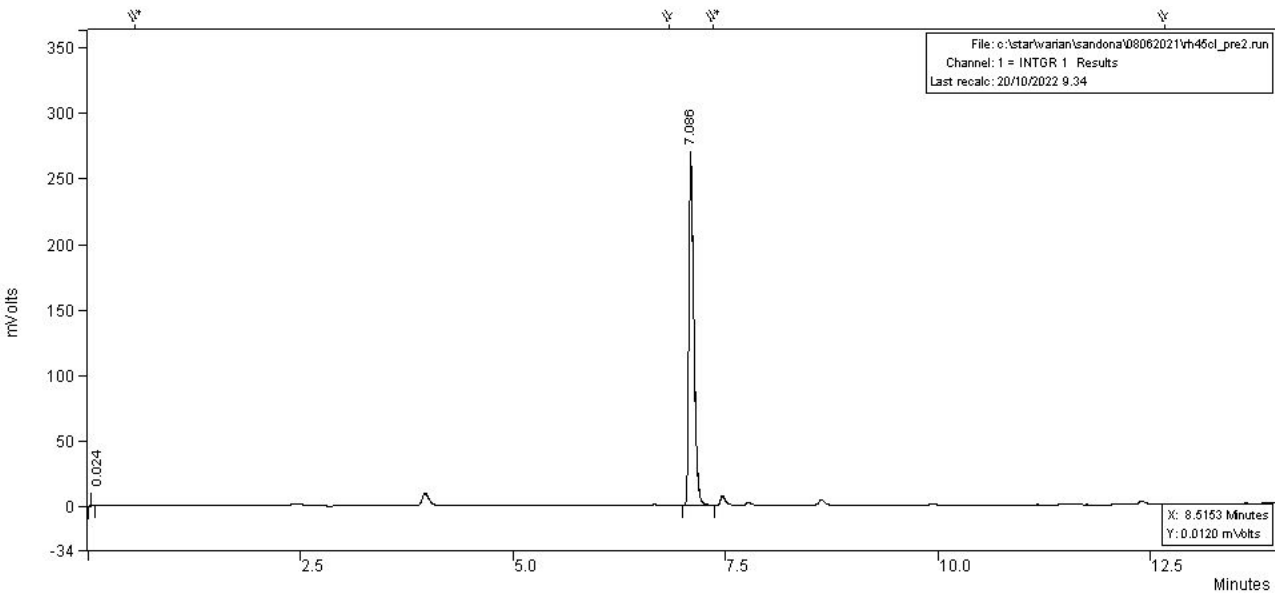

Figure S4. HPLC-UV profile (254 nm) of compound **5**.

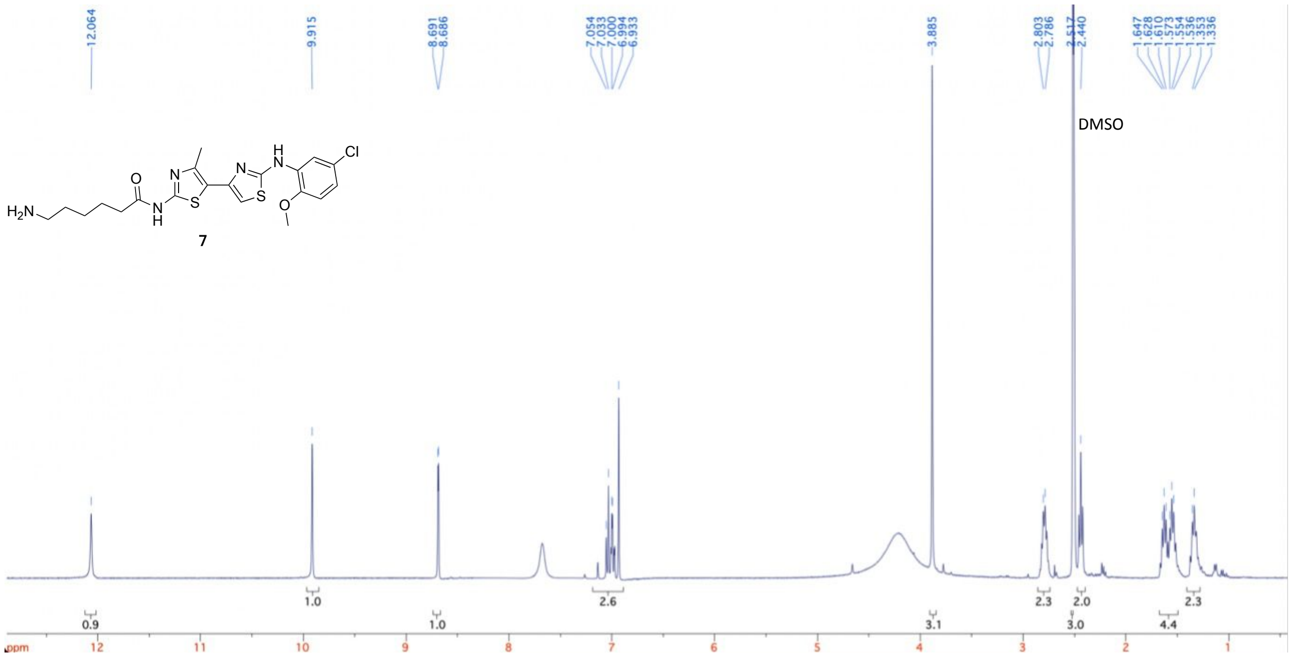

Figure S5. 1H-NMR of compound 7 (DMSO-d_6). Two exchangeable protons were not identified and included in the list of integrated signals.

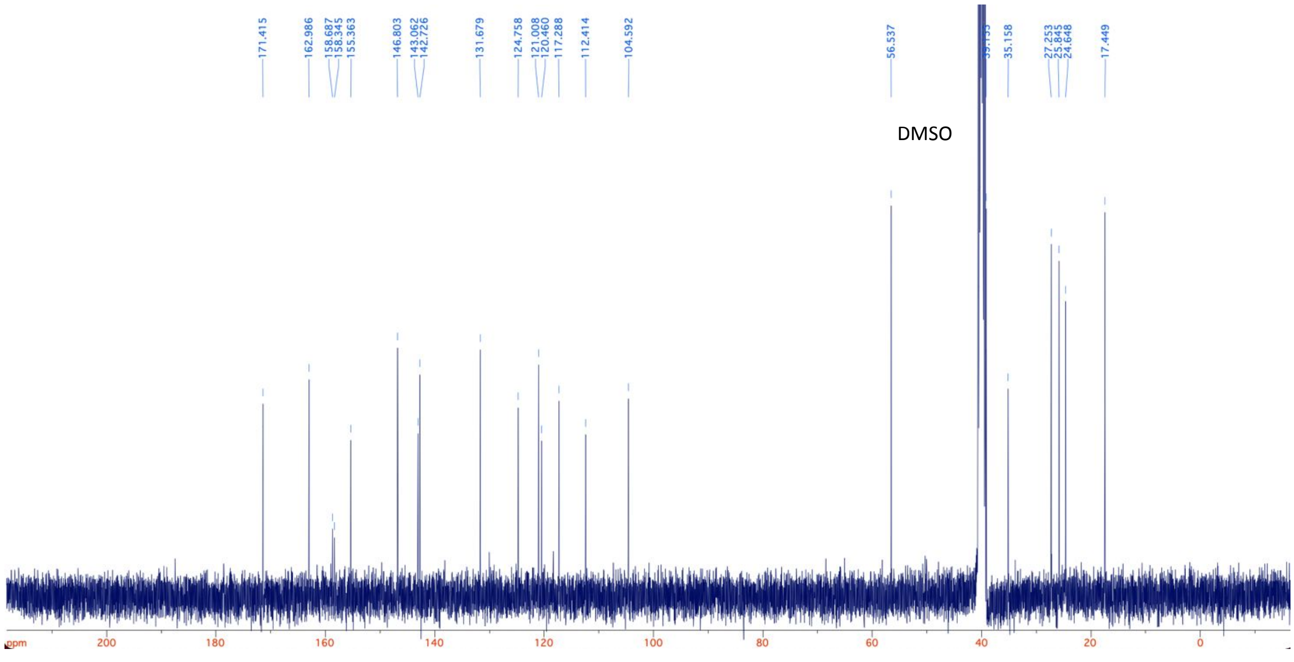

Figure S6. ^{13}C-NMR of compound **7** (DMSO-d_6).

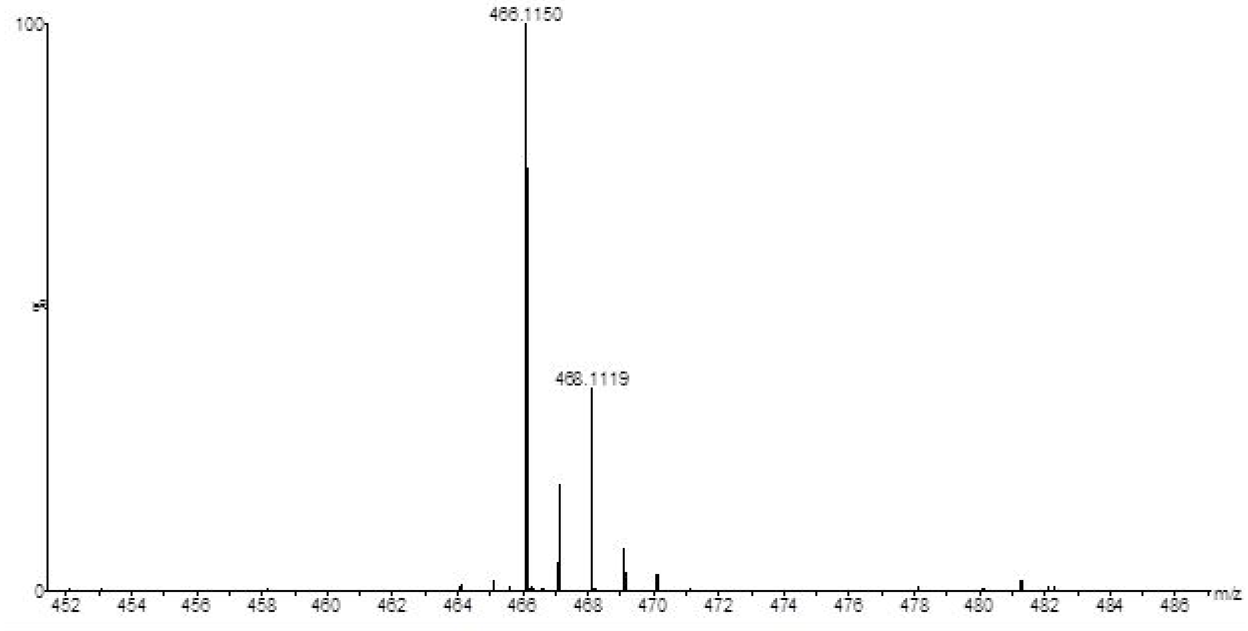

Figure S7. ESI-MS spectrum of compound **7** (positive ionization mode).

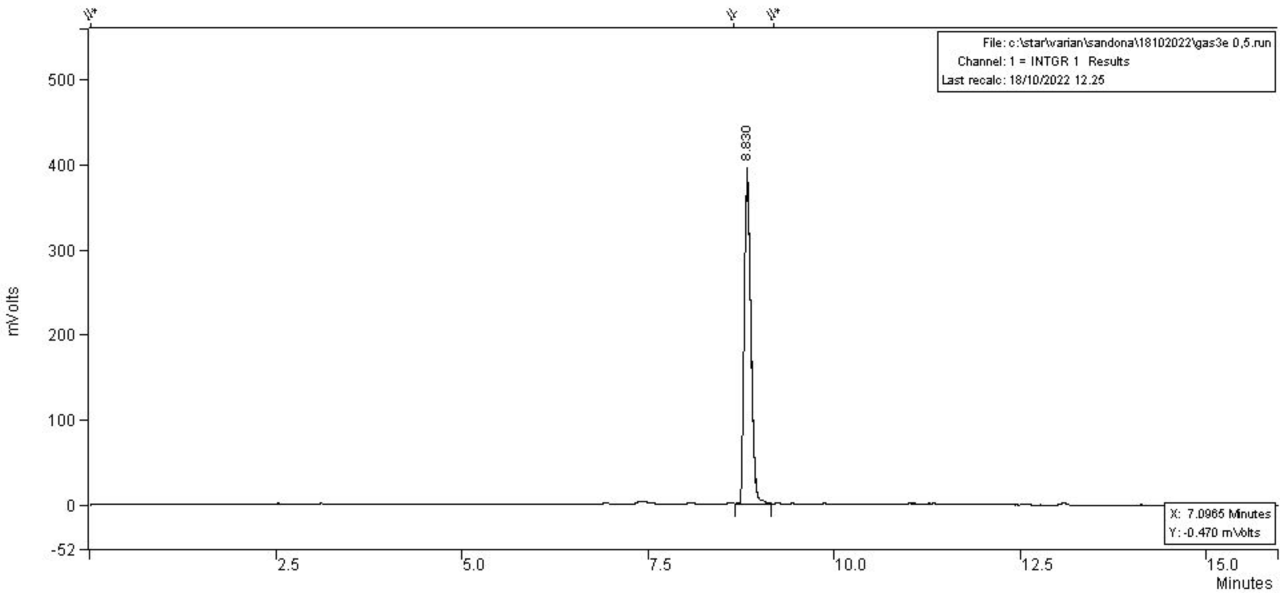

Figure S8. HPLC-UV profile (254 nm) of compound **7**.

Computational studies

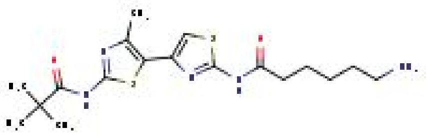

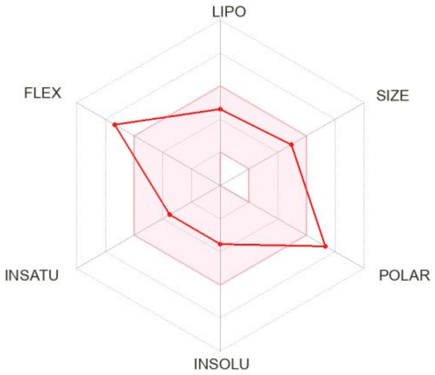

Compound **5**

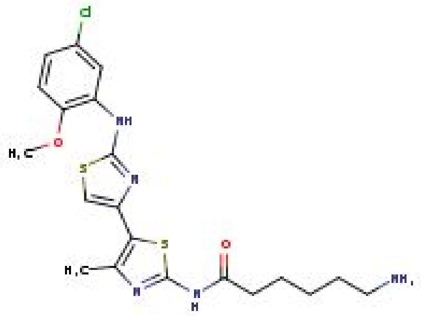

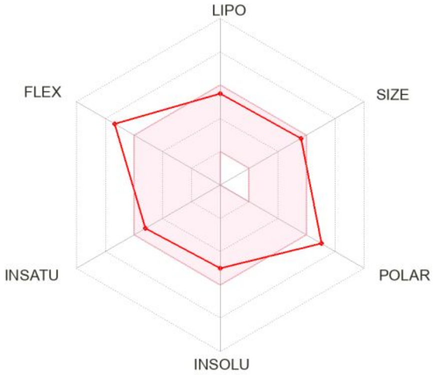

Compound **7**

Figure S9. Overview of the predicted physicochemical descriptors for compounds 5 and 7. The red area in the radar graphs represents the suitable physicochemical space for oral bioavailability, according to lipophilicity, size, polarity, solubility, insaturation and flexibility scores of the molecule.

Western blot analysis

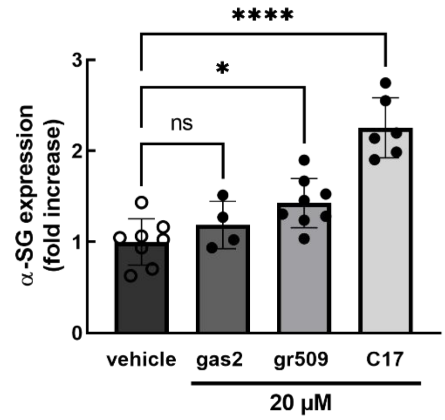

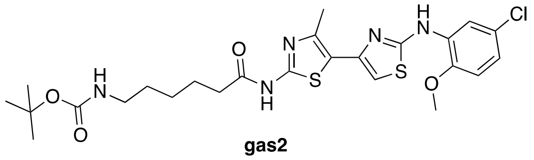

gas2

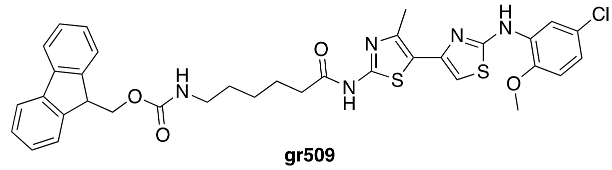

gr509

Figure S10. Additional in vitro studies. Densitometric analysis of independent WB experiments performed with total protein lysates from LGMDR3 myogenic cells carrying the L31P/V247M α-SG mutations, differentiated for 7 days and treated for the last 72 hours as indicated; vehicle (DMSO 1‰). Compounds named **gas2** and **gr509** represent the Boc- and Fmoc-protected precursors of compound **7**, respectively. The α-sarcoglycan (α-SG) protein was revealed by using specific primary antibody and normalized by the content of β-actin. The α-SG content is expressed as fold increase of the amount present in the vehicle treated myotubes, the mean value ± SD of a minimum of 4 independent experiments is also reported. Statistical analysis was performed by One-way ANOVA test followed by multiple comparisons Dunnett's test; n.s., $p > 0.05$; *, $p \leq 0.05$; ****, $p \leq 0.0001$.

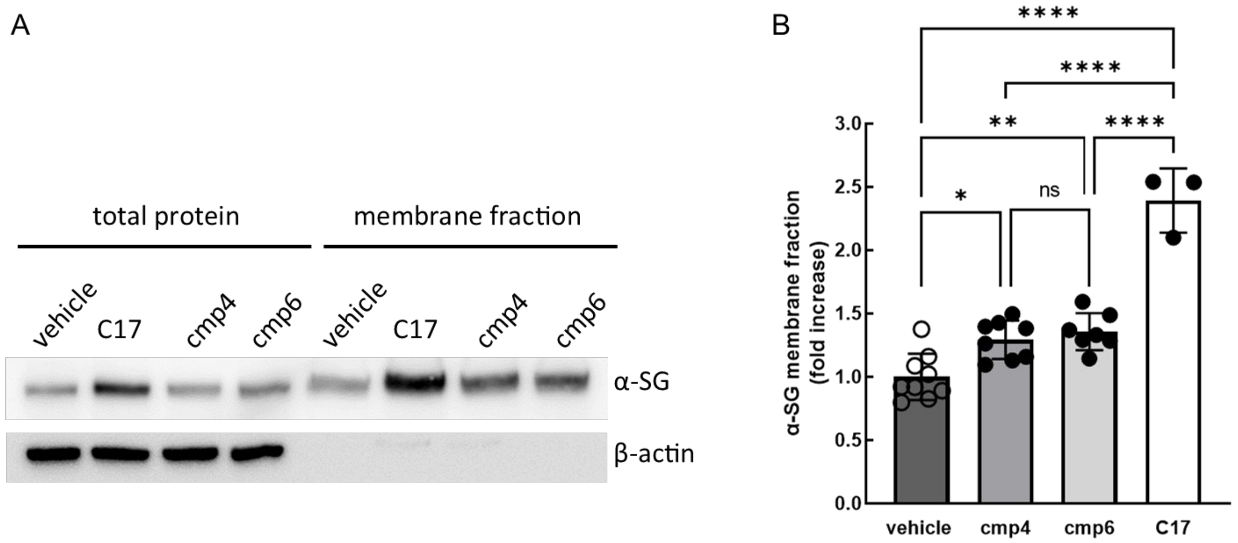

Figure S11. Analysis of the fraction of α-sarcoglycan (α-SG) present at the membrane of LGMDR3 myogenic cells. Myogenic cells from a LGMDR3 subject carrying the L31P/V247M α-SG mutations were differentiated for 7 days and treated for the last 72 hours with either DMSO 1‰ (vehicle), compound 4, compound 6 (cmp) or C17 all at 20 μM. At the end of incubation, myotubes were subjected to biotinylation and pull down with streptavidin-conjugated agarose beads, as described in Material and Methods. A) Biotinylated-surface proteins were analyzed by WB using antibodies specific for α-SG and β-actin, the latter used to check the absence of biotin internalization. In the same blot total protein lysates, input for the biotinylation assay, were also loaded (total protein). In this case β-actin served as normalizer of protein loading. B) the graph reports the quantification of the membrane fraction of α-SG performed by densitometric analysis of WB experiments. The α-SG content is expressed as the fold increase of the amount present in the vehicle treated myotubes. The mean value ± SD of minimum 3 independent experiments is reported. Statistical analysis was performed by One-way ANOVA test followed by multiple comparison Tukey's test; ns, $p > 0.05$, *, $p \leq 0.05$, **, $p \leq 0.01$, ****, $p \leq 0.0001$.

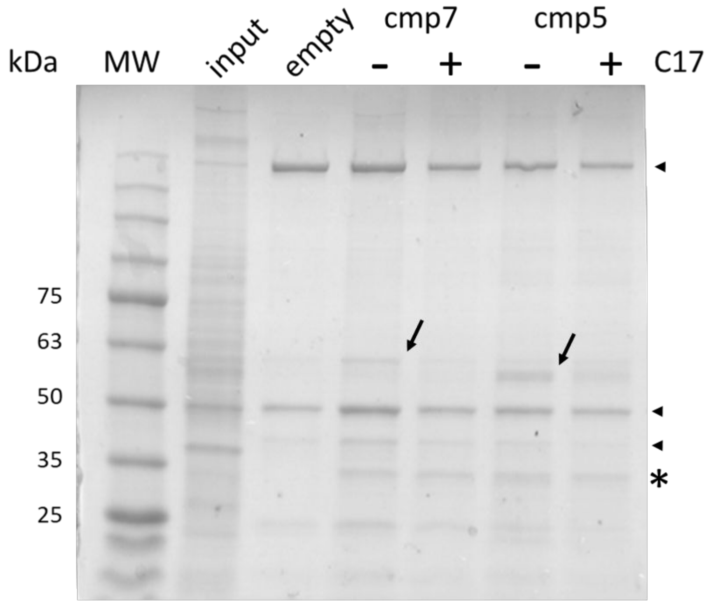

Figure S12. PoC of affinity chromatography experiments carried out with functionalized cmp7 and cmp5. Myogenic cells from a LGMDR3 subject carrying the L31P/V247M α-SG mutations were differentiated for 7 days. Myotubes were then incubated with T-PER (a lysis buffer commonly used in immunoprecipitation assays) to lysate cells preserving protein-protein interactions. After quantification, the same amount of protein lysates was incubated overnight with the different resins under gentle rotation, at 4°C in the presence (+) or absence (-) of an excess of free C17 compound. After extensive washing, bound proteins were eluted by the addition of the Laemmli sample Buffer, and loaded in the gels. Coomassie blue staining allowed the visualization of the protein bands. Some bands were common to both functionalized and empty resins (arrowed), suggesting an unspecific binding of polypeptides to the beads, another band is recovered by both functionalized resins (asterisk), while others protein bands (arrows) seem to have been recovered exclusively by cmp7 or cmp5 functionalized resin. The interaction also appears to be specific, as incubation with an excess of the free parent compound (C17) causes the displacement of the protein interactors from the resins. Total protein lysate from myotubes (input) was loaded for reference; MW, protein molecular weight markers.
